# Rice Bran Fermentation Using *Lactiplantibacillus plantarum* EM as a Starter and the Potential of the Fermented Rice Bran as a Functional Food

**DOI:** 10.3390/foods10050978

**Published:** 2021-04-29

**Authors:** Song-Hee Moon, Hae-Choon Chang

**Affiliations:** Kimchi Research Center, Department of Food and Nutrition, Chosun University, 309 Pilmun-daero, Dong-gu, Gwangju 61452, Korea; dal_moon@chosun.ac.kr

**Keywords:** *L. plantarum* EM, rice bran fermentation, cholesterol removal, antimicrobial activity, sensory quality

## Abstract

Rice bran was fermented using a functional starter culture of *Lactiplantibacillus plantarum* EM, which exhibited high cholesterol removal and strong antimicrobial activity. Highest viable cell counts (9.78 log CFU/mL) and strong antimicrobial activity were obtained by fermenting 20% rice bran supplemented with 1% glucose and 3% corn steep liquor (pH 6.0) at 30 °C for 48 h. The fermented rice bran slurry was hot air-dried (55 °C, 16 h) and ground (HFRB). HFRB obtained showed effective cholesterol removal (45–68%) and antimicrobial activities (100–400 AU/mL) against foodborne pathogenic bacteria and food spoilage fungi. Phytate levels were significantly reduced during fermentation by 53% due to the phytase activity of *L. plantarum* EM, indicating HFRB does not present nutrient deficiency issues. In addition, fermentation significantly improved overall organoleptic quality. Our results indicate that HFRB is a promising functional food candidate. Furthermore, HFRB appears to satisfy consumer demands for a health-promoting food and environmental and legal requirements concerning the re-utilization of biological byproducts.

## 1. Introduction

Rice bran is produced during rice milling and is one of the most abundant agricultural byproducts. Most of the rice bran produced is used as an animal feed ingredient and to produce fertilizers due to its poor taste and smell [1]. However, rice bran is rich in dietary fiber, protein, minerals, and phytochemicals, which are important health-promoting food ingredients [1,2]. Thus, to enhance the nutritional qualities of rice bran, many food processing techniques such as fermentation using fungi or extraction with different solvents have been used [3,4,5,6]. In particular, fermentation has been used to improve sensory qualities, enhance nutritional qualities, and reduce undesirable compound levels [7]. Specifically, for rice bran fermentation, most investigators have adopted solid-state fermentation using non-toxigenic filamentous fungi, such a *Rhizopus* and/or *Aspergillus* strains, as starter cultures to develop prebiotic, antioxidant or preservative, or to enhance cosmeceutical properties [2,3,4,5]. Most bacterial rice bran fermentations described have been carried out using lactic acid bacteria (LAB) to produce useful compounds (lactic acid or ornithine), to improve sensory characteristics, or to enhance LAB viabilities or phytochemical levels [8,9,10,11,12,13]. Generally, fungi grow well in rice bran cultures without additional nutrients, but LAB are difficult to grow in rice bran, as they have complex nutrient requirements, which include amino acids, vitamins, fatty acids, purines, and pyrimidines [12,14]. Thus, other investigations on rice bran fermentation using LAB used enzyme hydrolyzed rice bran, rice bran extract, or rice bran supplemented with other nutrients such as milk, yeast extract, soybean hydrolysate, arginine, or whey [9,12,13,14]. However, the utilization of rice bran as a functional food is limited despite its potential as a rich source of valuable health-promoting compounds.

Hypercholesterolemia is strongly associated with coronary heart disease, which is an important cause of death [7]. Several LAB strains have been reported to have cholesterol-lowering effects in vitro or in vivo [7]. We also reported on the cholesterol-lowering effect of *Lactobacillus plantarum* EM isolated from kimchi [15]. *Lactobacillus plantarum* was renamed *Lactiplantibacillus plantarum* according to a reclassification of the genus *Lactobacillus* in 2020 [16]; thus, we refer to it throughout as *Lactiplantibacillus plantarum*. *Lactiplantibacillus plantarum* EM showed high cholesterol removal by growing cells and even dead bacterial cells. Cholesterol removal mechanisms by *L. plantarum* EM were verified to enzymatic assimilation including bile salt hydrolase assimilation and cell surface-binding. *L. plantarum* EM also appeared to meet the functional criteria required for health-promoting probiotics, including acid and bile tolerance and antibiotic susceptibility [15]. Moreover, *L. plantarum* EM exhibited strong antimicrobial activities against different foodborne pathogenic bacteria and food spoilage fungi, and the active compounds involved were identified as 3-hydroxy-5-dodecenoic acid and lactic acid [17]. Cabbage-apple juice was also fermented using *L. plantarum* EM as a functional starter culture, and the fermented juice obtained exhibited significant hypocholesterolemic and anti-obesity effects in rats [7,18].

Recently, environmental and legal pressures have forced the food and agricultural industries to find means of re-utilizing biological byproducts [2]. As consumer’s interests in the health benefits of food continue to increase, functional LAB starter culture-based fermentation has attracted attention as a means of producing food materials containing a wide range of health-promoting compounds. We considered rice bran fermentation using a functional starter culture such as *L. plantarum* EM might satisfy the demands of consumers and meet environmental and legal requirements.

In this study, rice bran was fermented using *L. plantarum* EM as a functional starter culture. We describe the optimization of fermentation conditions with respect to *L. plantarum* EM growth and antimicrobial activity, and the fermented bran produced was hot air-dried to produce a fermented rice bran product, which was evaluated for its functional and organoleptic properties to assess its potential as a functional food candidate.

## 2. Materials and Methods

### 2.1. Microbial Cultures and Media

Microorganisms and culture media used in this study are listed in Table S1. LAB were cultivated in de Man Rogosa Sharpe (MRS; Difco, Sparks, MD, USA) broth. Non-LAB bacteria were cultivated in Luria-Bertani (LB, Difco) agar. Molds were grown on malt extract agar (MEA, Difco) or potato dextrose agar (PDA, Difco). Strains were purchased from the American Type Culture Collection (ATCC; Manassas, VA, USA). The LAB *L. plantarum* EM and *Weissella koreensis* DB1 were isolated from kimchi, as we previously described [13,15].

### 2.2. Optimization of Rice Bran Fermentation Conditions

To prepare a starter culture, *L. plantarum* EM was cultivated in MRS broth at 30 °C for 24 h, centrifuged (9950× *g*, 5 min, 4 °C), washed with sterile distilled water, and resuspended in sterile distilled water containing the same volume of culture. Thereafter, the prepared starter culture was inoculated (1%; equivalent to ~7.0 log CFU/mL) in rice bran slurry as described in Section 2.2.1, Section 2.2.2 and Section 2.2.3 and fermented at 30 °C for 48 h.

To prepare rice bran filtrate, fermented rice bran slurry was filtered through four layers of sterile thin cloth, and filtrates were tested for pH, viable LAB cell count, and antimicrobial activities [13].

#### 2.2.1. Rice Bran Concentration in Rice Bran Slurry

Rice bran slurries containing 5, 10, 15, 20, 25, or 30% (*w*/*v*) of rice bran powder (Henanum Co., Yangpyeong, Korea) in distilled water (Table S2) were autoclaved (121 °C, 15 min), immediately cooled (20 °C), and viscosities were determined using different spindle speeds of 12–60 rpm using a viscometer (AMETEK Brookfield, Middleboro, MA, USA) equipped with a No. 4 spindle.

#### 2.2.2. Rice Bran Slurry Supplementation

To investigate the effects of different nutrients on rice bran fermentation, different nutrients including carbon (glucose, maltose, sucrose, or fructose), nitrogen (peptone, soytone, beef extract, or yeast extract), and/or complex compound sources (corn steep liquor; CSL) at different concentrations (1–5%) were added to rice bran slurry containing 20% rice bran powder (Table S2).

#### 2.2.3. pH and Temperature

To investigate the effect of temperature, *L. plantarum* EM was cultivated at 24, 30, or 37 °C for 24 h or 48 h in pH non-adjusted rice bran slurry. To investigate the effect of pH, *L. plantarum* EM was cultivated in rice bran slurry consisting of 20% rice bran with 3% CSL and 1% glucose at pH values ranging from 4.0 to 8.0 for 24 h or 48 h at 30 °C. Thereafter, viable cells [13] and antimicrobial (antibacterial and antifungal) activities [17] were determined.

#### 2.2.4. Viable Cells and pH

To investigate the effects of rice bran concentration and other nutrients on the cell growth and antimicrobial activity of *L. plantarum* EM, *L. plantarum* EM was inoculated (7.0 log CFU/mL) into each prepared rice bran slurry and cultivated at 30 °C for 48 h, after which viable cells were counted. Viable cell counts in fermented rice bran were counted by plating on MRS agar [13]. pH values were determined using a pH meter (Fisher, Hanover Park, IL, USA).

#### 2.2.5. Antimicrobial Activities

Antimicrobial activities were determined using spot-on-the-lawn assays [17]. To prepare MRS cultures, *L. plantarum* EM was cultivated in MRS broth for 24 h at 30 °C. Prepared MRS broths of *L. plantarum* EM and rice bran filtrates were centrifuged (9950× *g*, 15 min, 4 °C) and filtered (0.45 μm; Millipore, Beverly, MA, USA). Prepared cell-free MRS filtrates and cell-free rice bran filtrates were analyzed for antimicrobial activities. Plates were prepared by adding mold spores (6.0 log spores/20 mL of MEA or PDA) to 1.5% bacto agar for the antifungal assay or by spreading bacterial cells (6.0 log CFU/mL) onto LB agar for the antibacterial assay. Thereafter, 10 μL aliquots of the sample were spotted onto prepared plates. Antimicrobial activity, expressed as arbitrary units (AU) per milliliter, was defined as the reciprocal of the highest dilution that produced an inhibitory zone towards sensitive microorganisms. Activities were calculated using (1000/d) D, where D is the dilution factor and d is dose (10 μL of prepared antimicrobial sample).

### 2.3. Preparation of Fermented Rice Bran

Under optimized fermentation conditions, rice bran was fermented at 30 °C for 48 h using *L. plantarum* EM as a starter culture, and hot air-dried at 55 °C for 16 h (Temperature & Humidity Chamber, HB-105SP, Hanbaek, Bucheon, Korea) to produce hot air-dried fermented rice bran (HFRB). Thereafter, it was ground using a blender (BW-3000, Buwon, Daegu, Korea) and tested for cholesterol removal, antimicrobial activity, phytic acid content, and organoleptic qualities. Raw rice bran (RRB) and hot air-dried non-fermented rice bran (HNRB) were used as controls.

### 2.4. Cholesterol Removal

Cholesterol removal by fermented rice bran was determined using the method devised by Rude and Morris [19]. To assay cholesterol removal, ~2 g of HFRB (obtained from 10 mL of fermented rice bran slurry) was resuspended in MRS broth containing 0.5% oxgall and 0.1 g/L of water-soluble cholesterol as well as 0.5% TDCA and 0.1 g/L of water-soluble cholesterol in 10 mL. Separately, RRB or HNRB slurries were also prepared. To prepare dead LAB cells, *L. plantarum* EM was incubated at 30 °C in MRS broth (10 mL) containing 0.5% oxgall or 0.5% TDCA and harvested (9950× *g*, 5 min, 4 °C). Cell pellets were suspended in saline and autoclaved at 121 °C for 15 min. Dead cells were harvested and then resuspended in MRS broth (10 mL) containing 0.5% oxgall and 0.1 g/L water-soluble cholesterol as well as 0.5% TDCA and 0.1 g/L water-soluble cholesterol [15]. 

Prepared resuspensions (10 mL) were incubated at 37 °C for 24 h in a GasPak EZ system (Becton Dickinson, Sparks, MD, USA) and harvested (9950× *g*, 4 °C, 5 min), and cholesterol concentrations in supernatants were determined as previously described [15]. Briefly, 1 mL of supernatant was added to 2 mL of 33% (*w*/*v*) potassium hydroxide and 3 mL of 95% (*v*/*v*) ethanol, heated for 10 min in a 60 °C water bath, and then cooled with tap water. Hexane (5 mL) was then added, mixed, and 1 mL of distilled water was added. After standing for 10 min to allow phase separation, the hexane phase was evaporated under a nitrogen stream. The concentrated aqueous fraction was then added to 4 mL of freshly prepared *ο*-phthalaldehyde (Sigma-Aldrich, St. Louis, MO, USA, 0.5 mg in 1 mL acetic acid), mixed, allowed to stand for 10 min, and treated with 2 mL of concentrated sulfuric acid for 10 min. Thereafter, absorbances were measured at 550 nm using an Ultrospec 2100 pro (Biochrom, Cambridge, UK). Cholesterol removal (%) was expressed as {0.1 g/L of cholesterol—concentration of remaining cholesterol in the cultures}/0.1 g/L of cholesterol ×100.

### 2.5. Phytase Activity and Phytic Acid

Phytase activity of *L. plantarum* EM was determined using phytase-specific medium as previously described [20]. Overnight grown *L. plantarum* EM was harvested (9950× *g*, 5 min, 4 °C), and suspended in the same volume of distilled water. *W. koreensis* DB1, which was used as a starter culture for rice bran fermentation in our previous study [13], and *L. plantarum* ATCC 14,917 were used as controls. Paper discs (diameter 8 mm; Advantec, Tokyo, Japan) were placed on phytase-specific medium (1.5% glucose, 0.5% calcium phytate, 0.5% NH_4_NO_3_, 0.05% KCl, 0.05% MgSO_4_·7H_2_O, 0.001% FeSO_4_·7H_2_O, 0.001% MnSO_4_, and 1.5% micro agar, pH 5.5), and 100 μL of prepared LAB suspensions was spotted onto the paper discs. Plates were incubated at 30 °C for 24–48 h, and the diameters of haloes formed on plates were measured using a caliper (Mitutoyo, Tokyo, Japan).

Total phytic acid contents of the rice bran products were determined using a phytic acid assay kit K-PHYT (Megazyme; Wicklow, Ireland) according to the manufacturer’s instructions. Briefly, the rice bran products (1 g) were extracted using 0.66 M hydrochloric acid and then neutralized with 0.75 M sodium hydroxide. Extracted samples were tested for total phosphorus and free phosphorus produced by phytase and alkaline phosphatase using the assay kit. Phytate contents were calculated using the following Equation (1):

(1) $$\mathrm{Phytate}\;\mathrm{content}\;\left(g/100\;g\right)=\frac{\mathrm{Phosphorus}\;\mathrm{content}\;\left(g/100\;g\right)}{0.282}$$

where, phosphorous content (g/100 g) = mean M × 0.1112 × ΔA phosphorous, ΔA phosphorous = Absorbance of total phosphorous − Absorbance of free phosphorous, and mean M (μg/ΔA phosphorous) = mean of phosphorous standard values (standard solutions 1–4 in the Megazyme K-PHYT kit).

### 2.6. Sensory Evaluations

Sensory evaluations were performed after obtaining approval from the Institutional Review Board of Chosun University (Gwangju, Korea; IRB#2-1041055-AB-N-01-2019-33). Nine trained students who had performed more than 30 evaluations per year at the Department of Food and Nutrition, Chosun University, constituted the sensory evaluation panel. Four samples of RRB, HNRB, HFRB, or HFRB containing 0.07% stevia (Viomix, Seoul, Korea; HFRB-S) were presented to the panel. Prior to sensory evaluations, panelists rinsed their mouths with water and waited for 1–2 min before performing each evaluation. Sensory attributes were evaluated using a 5-point scale for sourness, bitterness, sweetness, hay smell, and pleasant flavor (1 = very weak, 3 = moderate, and 5 = very strong) and mouthfeel texture and overall acceptability (1 = very bad, 3 = moderate, and 5 = very good).

### 2.7. Statistical Analysis

Data were expressed as the mean and standard deviation (mean ± SD) of triplicate determinations. Experimental data were statistically analyzed by Duncan’s multiple range test for one-way ANOVA in SPSS 26.0 software, and *p* < 0.05 was considered statistically significant.

## 3. Results and Discussion

### 3.1. Rice Bran Fermentation

#### 3.1.1. Rice Bran as a Nutrient Source for LAB Cell Growth

High *L. plantarum* EM cell growth during rice bran fermentation is most important to obtain in terms of developing a functional rice bran product, as *L. plantarum* EM, whether alive or dead, has shown high cholesterol removal ability [15]. *L. plantarum* EM was inoculated (7.00–7.04 log CFU/mL) in rice bran slurries (5–30% rice bran powder in distilled water) and cultivated at 30 °C for 24–48 h. Highest viable cell counts were obtained at 24 h (8.30–8.67 log CFU/mL), and these were maintained at 48 h. Viable cell counts were not affected by rice bran concentration (5–30%) (Table 1). The viscosities of 25–30% rice bran slurries could not be determined because they were almost solid. However, 20% of slurry viscosities ranged from 5733 to 17,514 cp at 12–60 rpm (data not shown); thus, we used this concentration for further experiments. The majority of previous studies have been conducted using solid-state fungal fermentations of 30–50% (*w*/*v*) rice bran slurries [2,3,4,5], whereas liquid-state rice bran fermentations of bacteria (LAB) have been performed using lower concentrations (<10% of rice bran or rice bran extract) [9,12,14].

The effects of other nutrients added to rice bran on LAB cell growth were also determined (Table 1). In carbon source supplementation to 20% rice bran, viable LAB cells of the cultures were 8.60–8.69 log CFU/mL. Whereas supplementation with 3% beef extract as a nitrogen source or 3% CSL as a complex compound source resulted in significantly better cell growth. Based on the above results, we decided that nutrients should be added to rice bran fermentations at 1% for glucose (as a primary carbon source), 3% CSL, and/or 3% beef extract. Two or three of these nutrients were used in combination, and then viable cell counts were determined. The highest viability (9.60–9.78 log CFU/mL) was obtained from 20% rice bran slurries containing 3% CSL supplemented with other nutrients (Table 1; 20% RB + combined nutrients); this cell growth was almost the same as that obtained (9.59 log CFU/mL) by MRS cultivation. In the present study, neither beef extract, peptone, soytone, nor yeast extract significantly affected the growth of *L. plantarum* EM, whereas CSL obviously increased *L. plantarum* EM growth. These results indicated that CSL addition is important to enhance LAB cell growth in rice bran culture. The cost of fermentation medium has been reported to account for almost 30% of the total cost of microbial fermentation [21]. CSL is a byproduct of corn wet-milling and contains carbon and nitrogen sources as well as minerals and vitamins. Thus, CSL has been proposed as an alternative to expensive nutrients such as beef extract, yeast extract, or peptone [21]. Behr suggested CSL be used as a nutrient for microorganisms as long ago as 1909 [22]. The usage of CSL as a component of nutrient media can be summarized as follows: Production of penicillin by mold, *Penicillium* sp. [22], production of ethanol, succinic acid, or arabinase by yeasts, *Zymomonas* sp. [23], *Pichia* sp. [24], *Anaerobiospirillum* sp. [25], or *Fusarium* sp. [26], and production of lactic acid by LAB [21,27,28,29]. However, its use in microbiology was limited to yeast fermentation until recently. Specifically, in studies on the fermentation using LAB, CSL has been used as an alternative for yeast extract to produce lactic acid [21,27,28,29]. However, results were less than satisfactory as CSL did not produce as much lactic acid as yeast extract [30]. In this study *L. plantarum* EM cell growth in rice bran culture was successfully enhanced byadding the biological byproduct CSL instead of expensive nutrients such as beef extract or yeast extract, which satisfies environmental and legal requirements concerning the re-utilization of biological byproducts. 

#### 3.1.2. Antimicrobial Activity of Fermented Rice Brans

MRS culture of *L. plantarum* EM (9.59 log CFU/mL) showed strong antifungal activity against *A. fumigatus* ATCC 96,918 (600 AU/mL) and antibacterial activity against *B. cereus* ATCC 14,579 (300 AU/mL) (Table 2). Rice bran cultures of *L. plantarum* EM (8.30–8.67 log CFU/mL) prepared from 5 to 30% rice bran/distilled water slurries did not exhibit antibacterial or antifungal activity. Rice bran cultures in rice bran slurries composed of 20% rice bran supplemented with different carbon or nitrogen sources showed antibacterial activity (100 AU/mL) against *B. cereus* ATCC 14,579 but no antifungal activity against *A. fumigatus* ATCC 96918. However, supplementation (>2% by weight) of CSL in rice bran slurry resulted in antibacterial (200 AU/mL) and antifungal (100 AU/mL) activities, and these antimicrobial activities were markedly higher than those of rice bran cultures supplemented with carbon or nitrogen but lower than those obtained by MRS culture. The addition of 3% CSL with glucose or/and beef extract increased the antifungal activity of fermented rice bran to 400 AU/mL, and the effect of beef extract supplementation on antimicrobial activity was similar to that of glucose. Supplementation with 3% CSL or 3% CSL + 1% glucose to rice bran culture both resulted in viable LAB cell counts of 9.70–9.78 CFU/mL, but the antifungal activity of rice bran culture supplemented with 3% CSL + 1% glucose was higher than that supplemented with 3% CSL (400 vs. 100 AU/mL) (Table 1). The results in Table 1 and Table 2 show supplementation with 3% CSL and 1% glucose achieved high *L. plantarum* EM cell growth (9.78 log CFU/mL) and strong antibacterial activity (200 AU/mL) against *B. cereus* ATCC 14,579 and antifungal activity (400 AU/mL) against *A. fumigatus* ATCC 96918. Notably, these results demonstrate that replacement of beef extract, peptone, soytone, or yeast extract with CSL resulted in higher LAB cell growth and antimicrobial activities. The antimicrobial mechanism was verified as *L. plantarum* EM with antimicrobial activity induced dimples on the surface of vegetative *B. cereus* cells, which resulted in cell death. *L. plantarum* EM showing antimicrobial activity also affected the cell membranes of *A. fumigatus* conidium and *B. cereus* endospore, which led to collapsed and shrunken morphologies resulting in cell death [17].

Based on these results, *L. plantarum* EM was cultivated in 20% rice bran slurry supplemented with 3% CSL + 1% glucose in subsequent experiments.

#### 3.1.3. Effects of pH and Temperature

Prior to adjustment, the pH of the prepared rice bran slurry composed of 20% rice bran + 3% CSL + 1% glucose was pH 5.6 ± 0.3. To investigate the effects of pH and temperature on the fermentation, pH values and temperatures of rice bran slurry were adjusted in ranges pH 4.0–8.0 and 25–37 °C, respectively, and then viable LAB counts and antibacterial and antifungal activity were measured (Table 3). Highest cell growth and antimicrobial activity were obtained at pH from 6.0 to 7.0 and at 30 °C. Based on these results, rice bran at pH 6.0 was fermented at 30 °C in further experiments.

### 3.2. Characterization of Rice Bran Products

We fermented rice bran using a functional starter culture *L. plantarum* EM, which exhibited effective cholesterol removal regardless of LAB viability coupled with strong antimicrobial activities against foodborne pathogenic bacteria and food spoilage fungi. To address the unsuitability of rice bran as a growth medium for LAB, others have used enzyme hydrolyzed rice bran, rice bran extract, or rice bran with other nutrient supplementations [8,9,10,11,12,13]. However, in this study highest LAB cell growth (9.78 log CFU/mL) and strong antimicrobial activity were obtained by simply fermenting 20% rice bran in the presence of 3% CSL and 1% glucose (pH 6.0) at 30 °C for 48 h.

For customer convenience and to extend shelf-life, fermented slurries must be further processed by a drying process such as freeze-drying or hot air-drying. We adopted a hot air-drying method, which is more cost-effective than freeze-drying. After the fermented rice bran had been hot air-dried, no viable LAB cells were detected. Advantages of the hot air-drying method used in this study are that the used starter culture *L. plantarum* EM can represent its bioactivities (cholesterol removal and antimicrobial activity) even in dead cells [15,17], and that the used substrate in this fermentation is a heat-stable cereal-based material, rice bran.

#### 3.2.1. Cholesterol Removal by Fermented Rice Bran Products

The effect of the hot air-dried fermented rice bran product (HFRB) on reducing cholesterol levels in vitro was examined (Table 4). In this assay, raw rice bran (RRB), hot air-dried non-fermented rice bran product (HNRB), and dead cells of *L. plantarum* EM were used as controls. In our previous study, *L. plantarum* EM showed high cholesterol removal regardless of cell viability, but cholesterol removal by growing cells (88.12%) was greater than that by dead cells (39.02%) in oxgall assay [15]. Meanwhile, it has been reported that bioactive compounds, including tocotrienols, γ-oryzanol, and dietary fiber, in rice bran have cholesterol-lowering effects [31]. As shown in Table 4, RRB and HNRB achieved 7.77–9.75% cholesterol removal, and dead cells of *L. plantarum* EM achieved 34.67–39.58% cholesterol removal, while HFRB attained 44.93–67.58% in oxgall and TDCA assays. Cholesterol removal by HFRB was slightly lower than that achieved by *L. plantarum* EM live cells (47.66–88.12% in oxgall and TDCA assays [15]). These results demonstrated that the cholesterol removal efficacy of rice bran was significantly enhanced by fermentation. We suppose these results were due to the synergistic effects of bioactive compounds in rice bran, the strong cholesterol-binding ability of *L. plantarum* EM cell walls, and the effects of unidentified compounds produced during fermentation.

#### 3.2.2. Antimicrobial Activity

As we previously reported, *L. plantarum* EM showed antimicrobial activities against various foodborne pathogenic bacteria and food spoilage molds [17]. The compounds showing antibacterial and antifungal activities were identified as 3-hydroxy-5-dodecenoic acid and lactic acid. Furthermore, the antimicrobial effects of these active compounds were found to act synergistically [17].

Cholesterol removal and antimicrobial (both antibacterial and antifungal) activity are distinctive and important bioactivities of *L. plantarum* EM. Fermented rice bran products intended to be used as functional food or ingredients should possess these bioactivities. Thus, in this study, we examined whether the fermented rice bran product produced by hot air-drying (at 55 °C for 16 h) retained antimicrobial activity. To determine the antimicrobial activity of HFRB, 2 g (equivalent to 10 mL of rice bran slurry) was resuspended in 10 mL of distilled water, allowed to stand for 4–5 h at 4 °C, and filtered. The antimicrobial activities of the filtrates obtained were then evaluated against various foodborne pathogens and food spoilage microorganisms (Table 5). The antimicrobial activities of RRB (pH 6.3), HNRB (pH 5.6), HFRB (pH 3.8), and MRS culture filtrate (pH 3.8) of *L. plantarum* EM were measured. HFRB showed the same or slightly less antimicrobial activity against foodborne pathogenic bacteria (200–400 AU/mL) and food spoilage fungi (100–400 AU/mL) than *L. plantarum* EM MRS filtrate (200–400 AU/mL antibacterial and 100–600 AU/mL antifungal), but no antimicrobial activity was observed for RRB or HNRB (Table 5). Fermented rice bran product and MRS culture of *L. plantarum* EM had similar cell counts (Table 1) and pH values, and lactic acid amounts detected in HFRB and MRS culture filtrates were similar (data not shown). The reason why the antimicrobial activities of MRS culture filtrate against *A. fumigatus*, *P. expansum*, and *B. cereus* were slightly higher than those of HFRB (in Table 5) is believed to be due to the sodium acetate present in MRS medium. Stiles et al. reported that sodium acetate, a basic component of commercial MRS medium, has microorganism-dependent antifungal effects [32].

These results showed that the fermented rice bran product even after hot air-drying (HFRB) retained strong antimicrobial activities against different foodborne pathogenic bacteria and food spoilage molds. The results suggest that the natural antimicrobial agent, HFRB, could be used as a functional food.

#### 3.2.3. Phytic Acid

As shown in Table S3, *L. plantarum* EM showed obvious phytase activity (18 mm clear zone on phytase-specific medium), while other LAB strains showed lower activities (9–12 mm clear zone).

Phytic acid (*myo*-inositol hexaphophoric acid) is found in the seeds of plants and has some beneficial properties, which include antioxidant, anticancer, cholesterol-lowering, and blood-sugar-lowering activities [33]. However, phytic acid also inhibits the absorptions of essential minerals such as iron, zinc, calcium, and magnesium in the digestive tract due to its ability to bind polycations and incorporate them in insoluble complexes. Thus, phytic acid is considered an antinutrient, especially in children and the elderly, in whom calcium absorption is important [33].

As shown in Table 6, phytic acid contents were 3.99 g per 100 g for HFRB, 7.93 g per 100 g for HNRB, and 8.48 g per 100 g for RRB; that is, *L. plantarum* EM phytase reduced phytic acid content by 53% during fermentation. It has been reported that the amount of phytic acid present in foods depends on the preparation methods used, such as milling, soaking, germinating, or fermentation [33]. In healthy people eating a balanced diet, the inhibitory effect of phytic acid on mineral absorption is minimal, and phytic acid consumption at 1000–2000 mg per day has not been associated with nutrient deficiencies [34]. Therefore, consumption of HFRB is unlikely to have any antinutritional effect.

#### 3.2.4. Sensory Evaluation

As shown in Table 7, HFRB had a pleasant flavor, a soft mouthfeel, and a strong sour taste. The strong hay smell and bitterness of RRB and HNRB were notably reduced by fermentation. The sourness of HFRB was attributed to low pH (3.80 due to 83,726 mg/kg of lactic acid; data not shown), but this was improved by adding only 0.07% by weight of stevia without affecting sweetness. The consumption of rice bran has been limited despite its health-promoting compound contents because of its smell, taste, and coarse texture [1]. This study shows that the organoleptic qualities of rice bran, except sourness, were significantly improved by *L. plantarum* EM fermentation, and that this sourness was easily managed by adding a calorie-free sweetener, such as stevia (HFRB-S).

## 4. Conclusions

In this study, the fermented rice bran product (HFRB) showed high cholesterol removal (45–68%) and antimicrobial activities (100–400 AU/mL) against foodborne pathogenic bacteria and food spoilage fungi. Levels of phytic acid, which has a combination of beneficial and antinutrient properties, were significantly reduced (from 8480 mg/100 g to 3990 mg/100 g) during fermentation, which showed the antinutritional aspects of HFRB were not a cause for concern. HFRB showed much better organoleptic qualities than RRB and HNRB, except sourness, though this was easily improved by adding a small amount (0.07% by weight) of stevia. Commercially successful functional food products must satisfy consumer requirements for health-promoting properties and good sensory properties, and the results of this study show that HFRB well meets these requirements. Thus, we conclude that HFRB produced using *L. plantarum* EM is a promising functional food candidate or ingredient that can be incorporated into foods as a natural antimicrobial agent and health-promoting material without adversely affecting flavor or texture. The most important aspect of this study is that it resulted in the low-cost production of a valuable functional food candidate or functional food ingredient from an inexpensive food byproduct. We are currently evaluating HFRB in animals to confirm its beneficial health effects in vivo.

## Figures and Tables

**Table 1:** Effect of rice bran and other nutrients on the cell growth of *L. plantarum* EM.

Culture		Viable Cells (log CFU/mL)
Nutrient	Concentration (%)	
MRS (control)		9.59 ± 0.13 ^A^
Rice bran (RB)	5	8.58 ± 0.13 ^a^
	10	8.39 ± 0.41 ^a^
	15	8.64 ± 0.39 ^a^
	20	8.67 ± 0.38 ^aB^
	25	8.36 ± 0.01 ^a^
	30	8.30 ± 0.01 ^a^
20% RB + Carbon-source		
Glucose	1	8.69 ± 0.16 ^aB^
	3	8.66 ± 0.10 ^a^
Maltose	1	8.62 ± 0.02 ^a^
	3	8.63 ± 0.05 ^a^
Sucrose	1	8.65 ± 0.12 ^a^
	3	8.60 ± 0.04 ^a^
Fructose	1	8.63 ± 0.05 ^a^
	3	8.62 ± 0.12 ^a^
20% RB + Nitrogen-source		
Peptone	1	8.86 ± 0.19 ^a^
	3	8.94 ± 0.06 ^ab^
Beef extract	1	8.79 ± 0.11 ^a^
	3	9.05 ± 0.03 ^aB^
Yeast extract	1	8.84 ± 0.12 ^a^
	3	8.76 ± 0.04 ^a^
Soytone	1	8.81 ± 0.04 ^a^
	3	8.89 ± 0.04 ^ab^
20% RB + Complex compound source		
Corn steep liquor (CSL)	1	9.29 ± 0.18 ^b^
	2	9.33 ± 0.11 ^b^
	3	9.70 ± 0.11 ^aA^
	4	9.38 ± 0.09 ^b^
	5	9.40 ± 0.09 ^b^
20% RB + Combined nutrients		
20% RB + 1% Glucose + 3% Beef extract		9.03 ± 0.28 ^b^
20% RB + 1% Glucose + 3% CSL		9.78 ± 0.06 ^aA^
20% RB +3% Beef extract + 3% CSL		9.60 ± 0.05 ^aA^
20% RB + 1% Glucose + 3% CSL + 3% Beef extract		9.65 ± 0.12 ^aA^

*L. plantarum* EM was cultivated in rice bran slurry at 30 ℃ for 48 h when viable cells were counted. Values are the mean ± SD of three independent cultivations. Different lowercase letters indicate significant differences (*p* < 0.05) between supplemented nutrients in the same column. Different uppercase letters indicate significant differences (*p* < 0.05) between cultures (indicated by shading).

**Table 2:** Effect of rice bran and other nutrients on the antimicrobial activities of *L. plantarum* EM.

Culture.		Antimicrobial Activity (AU/mL)	
Nutrient	Concentration (%)	Antibacterial	Antifungal
MRS (control)		300	600
Rice bran (RB)	5	0	0
	10	0	0
	15	0	0
	20	0	0
	25	0	0
	30	0	0
20% RB + Carbon-source			
Glucose	1	100	0
	3	100	0
Maltose	1	100	0
	3	100	0
Sucrose	1	100	0
	3	100	0
Fructose	1	100	0
	3	100	0
20% RB + Nitrogen-source			
Peptone	1	100	0
	3	100	0
Beef extract	1	100	0
	3	100	0
Yeast extract	1	100	0
	3	100	0
Soytone	1	100	0
	3	100	0
20% RB + Complex compound source			
Corn steep liquor (CSL)	1	100	100
	2	200	100
	3	200	100
	4	200	100
	5	200	100
20% RB + Combined nutrients			
20% RB + 1% Glucose + 3% Beef extract		100	0
20% RB + 1% Glucose + 3% CSL		200	400
20% RB + 3% Beef extract + 3% CSL		200	400
20% RB + 1% Glucose + 3% CSL + 3% Beef extract		200	400

Rice bran slurry was fermented at 30 °C for 48 h, after which antimicrobial activities were assayed on *B. cereus* ATCC 14,579 and *A. fumigatus* ATCC 96,918 lawn plates as described in Materials and Methods.

**Table 3:** Effect of pH and temperature on cell growth and the antimicrobial activities of *L. plantarum* EM.

Factor	Viable Cells (CFU/mL)		Antimicrobial Activity (AU/mL)			
			Antibacterial		Antifungal	
	24 h	48 h	24 h	48 h	24 h	48 h
Temp. (°C) *						
25	9.49 ± 0.08 ^b^	9.28 ± 0.25 ^a^	100	200	100	200
30	9.78 ± 0.11 ^a^	9.33 ± 0.21 ^a^	200	200	200	400
37	9.47 ± 0.06 ^b^	8.69 ± 0.09 ^b^	100	200	100	200
pH **						
4.0	9.39 ± 0.10 ^c^	8.90 ± 0.30 ^cd^	100	100	100	100
5.0	9.44 ± 0.17 ^c^	9.22 ± 0.15 ^bc^	100	200	100	100
6.0	9.81 ± 0.10 ^a^	9.59 ± 0.07 ^a^	200	200	200	400
7.0	9.76 ± 0.13 ^ab^	9.49 ± 0.16 ^ab^	200	200	200	400
8.0	9.53 ± 0.14 ^bc^	8.70 ± 0.14 ^d^	100	200	100	200

* Rice bran slurries were incubated at 25, 30, or 37 ℃ without pH adjustment. ** pH values of rice bran slurries were adjusted to 4.0–8.0 and then incubated at 30 ℃. Different letters (a–d) represent significant differences (*p* < 0.05) on the same factor at 24 h and 48 h, respectively.

**Table 4:** Cholesterol removal by the fermented rice bran products.

Sample	Cholesterol Removal (%)	
	0.5% Oxgall	0.5% TDCA
Dead cells of *L. plantarum* EM	39.58 ± 0.49 ^b^	34.67 ± 1.34 ^b^
RRB	8.87 ± 3.77 ^c^	7.77 ± 1.33 ^c^
HNRB	9.75 ± 1.06 ^c^	9.29 ± 3.10 ^c^
HFRB	67.58 ± 3.34 ^a^	44.93 ± 1.21 ^a^

Rice bran slurry consisting of 20% rice bran powder (RRB) + 3% CSL + 1% glucose in distilled water (pH 6.0) was fermented using *L. plantarum* EM for 0 h (HNRB) or 48 h (HFRB) at 30 °C, hot air-dried at 55 °C, and then ground. Separately, *L. plantarum* EM was cultivated in MRS containing 0.5% TDCA or 0.5% oxgall at 37 °C for 24 h. Cell pellets were harvested and suspended in saline and heat-killed at 121 °C for 15 min. Prepared samples of RRB, HNRB, HFRB, and of the dead cells of *L. plantarum* EM were incubated at 37 °C for 48 h in 0.5% oxgall or 0.5% TDCA containing cholesterol media as described in Materials and Methods. Values in the same column with different letters indicate significant difference (*p* < 0.05).

**Table 5:** Antimicrobial activity of the fermented rice bran products.

Indicator Strains		Antimicrobial Activity (AU/mL)			
		RRB	HNRB	HFRB	MRS Culture Filtrate *
Molds	*Aspergillus flavus* ATCC 22546	0	0	200	200
	*Aspergillus fumigatus* ATCC 96918	0	0	400	600
	*Penicillium roqueforti* ATCC 10110	0	0	100	100
	*Penicillium expansum* ATCC 7861	0	0	0	100
Bacteria	*Bacillus cereus* ATCC 14579	0	0	200	300
	*Escherichia coli* O157:H7 ATCC 43895	0	0	200	200
	*Pseudomonas aeruginosa* ATCC 29853	0	0	400	400
	*Salmonella enterica* servoar. Typhi ATCC 14028	0	0	200	200

Rice bran slurry consisting of 20% rice bran powder (RRB) + 3% CSL + 1% glucose in distilled water (pH 6.0) was fermented using *L. plantarum* EM for 0 h (HNRB) or 48 h (HFRB) at 30 °C, hot air-dried (55 °C), and ground. Products were resuspended in distilled water, and cell-free filtrates were prepared to assay antimicrobial activities, as described in Materials and Methods. * To prepare MRS culture filtrate, *L. plantarum* EM was cultivated in MRS broth at 30 °C for 48 h, centrifuged, and filtered.

**Table 6:** Phytic acid content of fermented rice bran products.

Sample	Phytic Acid (g/100 g)
RRB	8.48 ± 0.47 ^a^
HNRB	7.93 ± 0.44 ^a^
HFRB	3.99 ± 0.26 ^b^

Values displayed with different letters are significantly different (*p* < 0.05).

**Table 7:** Results of sensory evaluations of fermented rice bran products.

Indicator Strains	RRB	HNRB	HFRB	HFRB-S
Sourness	1.11 ± 0.33 ^d^	2.22 ± 0.44 ^c^	4.67 ± 0.50 ^a^	3.89 ± 0.78 ^b^
Bitterness	3.33 ± 0.71 ^b^	4.11 ± 0.33 ^a^	2.00 ± 0.00 ^c^	2.00 ± 0.00 ^c^
Sweetness	2.00 ± 0.00 ^a^	2.00 ± 0.00 ^a^	2.00 ± 0.00 ^a^	2.00 ± 0.00 ^a^
Hay smell	5.00 ± 0.00 ^a^	4.00 ± 0.00 ^b^	1.67 ± 0.71 ^c^	1.67 ± 0.71 ^c^
Pleasant flavor	1.78 ± 0.83 ^c^	2.78 ± 0.83 ^b^	4.89 ± 0.33 ^a^	4.89 ± 0.33 ^a^
Mouthfeel texture	1.56 ± 0.53 ^c^	2.67 ± 0.50 ^b^	4.11 ± 0.60 ^a^	4.11 ± 0.33 ^a^
Overall acceptability	1.67 ± 0.50 ^c^	2.00 ± 0.00 ^c^	3.00 ± 0.50 ^b^	4.11 ± 0.60 ^a^

Rice bran slurry was fermented at 30 °C for 0 h (HNRB) or 48 h (HFRB), hot air-dried at 55 °C for 14 h, and then ground. Sensory evaluations were carried out on raw rice bran (RRB), fermented rice bran product (HFRB), non-fermented rice bran product (HNRB), and HFRB containing 0.07% by weight of stevia (HFRB-S). Evaluations were performed using a 5-point scale; 1, 3, and 5 corresponding to “very bad, moderate, and very good” for mouthfeel texture and overall acceptability, and using “very weak, moderate, and very strong” for the other items. Results are expressed as the mean ± SD. Different letters for sensory items indicate significant differences (*p* < 0.05).

## Data Availability

Data is contained within the article or supplementary material.

## Supplementary Materials

The following are available online at https://www.mdpi.com/article/10.3390/foods10050978/s1, Table S1: Microorganisms used in this study, Table S2: Rice bran slurry supplemented with different nutrients, Table S3: Phytase activity of *L. plantarum* EM.
