# Supplementary material for: Rice Bran Fermentation Using Lactiplantibacillus plantarum EM as a Starter and the Potential of the Fermented Rice Bran as a Functional Food
Source: Foods. 2021 Apr 29;10(5):978. doi: 10.3390/foods10050978 (PMC8144953; doi:10.3390/foods10050978)
Supplement: Supplementary file 1 [file foods-10-00978-s001.zip › foods-1150651-supplementary.pdf]

Table S1. Microorganisms used in this study.

Miroorganism	Medium	Culture condition
**LAB**		
Lactobacillus plantarum EM	MRS	30℃ or 37℃, 24 h
Weissella koreensis DB1	MRS	30℃, 24 h
Lactobacillus plantarum ATCC 14917	MRS	30℃, 24 h
**Bacteria**		
Bacillus cereus ATCC 14579	LB	37℃, 24 h
Escherichia coli O157:H7 ATCC 43895	LB	37℃, 24 h
Pseudomonas aeruginosa ATCC 27853	LB	37℃, 24 h
Salmonella enterica serovar. Typhi ATCC 14028	LB	37℃, 24 h
**Molds**		
Aspergillus flavus ATCC 22546	MEA	30℃, 48 h
Aspergillus fumigatus ATCC 96918	MEA	30℃, 48 h
Penicillium roqueforti ATCC 10110	PDA	25℃, 48 h
Penicillium expansum ATCC 7861	PDA	25℃, 48 h

MEA; malt extract agar, PDA; potato dextrose agar, LB: Luria-Bertani, MRS: de Man Rogosa Sharpe

Table S2. Rice bran slurry supplemented with different nutrients.

Rice bran slurry	Supplemented nutrients and their concentrations used
Basic slurry : 20 % rice bran powder distilled in water	+ None<br>+ Carbon source: 1 and 3% (w/v) of glucose, maltose, sucrose, or fructose<br>+ Nitrogen source: 1, 3, and 5% (w/v) of peptone, soytone, beef extract, or yeast extract<br>+ Complex compound source: 1, 2, 3, 4, and 5% (w/v) of corn steep liquor (CSL)<br>+ Combined nutrients: 1% of glucose + 3% beef extract,<br>1% of glucose + 3% CSL,<br>1% of glucose + 3% of beef extract + 3% of CSL<br>3% of beef extract + 3 % CSL, or<br>1% of glucose + 3% of beef extract + 3% of CSL

Table S3. Phytase activity of *L. plantarum* EM.

LAB strains	Diameter of clear zone (mm)
Lactobacillus plantarum EM	18.14±0.10[a]
Lactobacillus plantarum ATCC 14917	12.51±0.14[b]
Weissella koreensis DB1	9.01±0.02[c]

Means ±SDs with different letters were significantly different ($p<0.05$) by Duncan's multiple range test.
